# Billateral sudden deafness

**DOI:** 10.1590/S1808-86942011000500026

**Published:** 2015-10-22

**Authors:** Therezita M. Peixoto Patury Galvão Castro, Laurisson Albuquerque da Costa, Maria Eliza Alencar Nemezio, Lucas José Sá da Fonseca

**Affiliations:** 1 PhD in Medicine - School of Medical Sciences of the Santa Casa de São Paulo (FCMSCSP). Adjunct Professor of Otorhinolaryngology - UNCISAL and UFAL; 2 6th year medical student - Federal University of Alagoas; 3 6th year medical student - Federal University of Alagoas; 4 6th year medical student - Federal University of Alagoas

**Keywords:** hearing loss, neurilemmoma, stroke, sudden

## INTRODUCTION

Sudden hearing loss is described as a sensorineural hearing loss starting at 30 decibels (dB) in three of more contiguous frequencies, which can be severe and irreversible, which onset happens suddenly along 3 days. Auditory involvement is usually unilateral in 98%-99% of the cases^1^,^2^. It does not have a well-defined etiological factor, and most are idiopathic; it can be due to vascular involvement, viral infection, autoimmune disorder or rupture of the intracochlear membrane; etiology confirmation is rather difficult, since many can be the causal agents. Moreover, these factors may manifest in a synergic way, giving the disorder a multifactorial etiology^3^,^4^. The acoustic neuroma is considered a definitive cause of sudden hearing loss. The most frequent accompanying symptoms are tinnitus in 70% to 90% of the cases and dizziness in 20% to 40% of the cases^4^,^5^.

For the sudden hearing loss diagnosis, besides the detailed anamneses and physical exam, a complete audiometric evaluation is paramount, brainstem evoked-response audiometry (BERA), vestibular tests and trigeminal nerve assessment, as well as MRI with gadolinium and other tests in the pursue of the etiology^5^,^6^.

## CASE PRESENTATION

**Figure 1:**
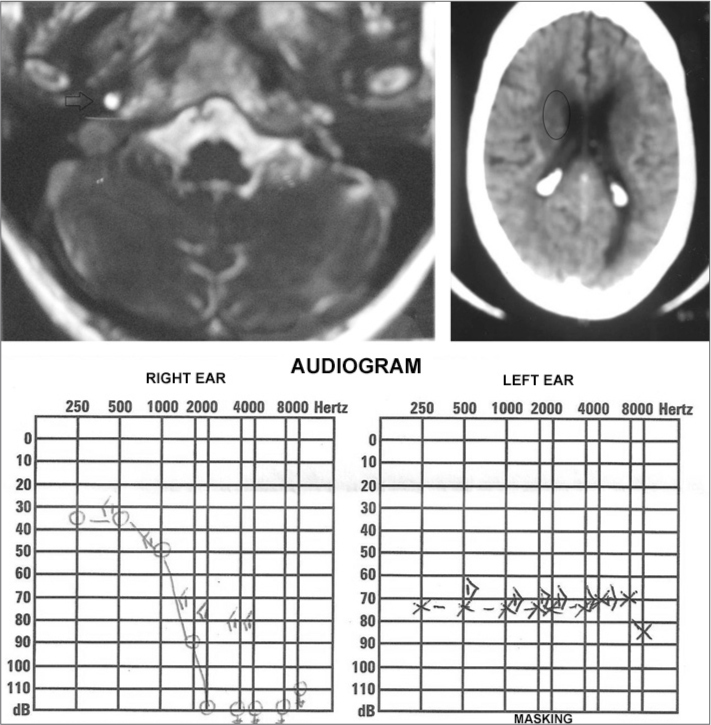
Complementary tests. Upper left-hand image: MRI showing an acoustic neuroma to the right (arrow); upper righthand image: skull CT showing a hypodense area near the right ventricle horn (circle); lower image: tonal audiometry showing bearing loss after a sudden hearing loss spell.

M.T.T.A., female, an 82 year-old retiree, coming from Maceió - AL, was seen in the ENT ward, where she complained of tinnitus and sudden hearing loss in her right ear for about 12 years, at the time she was diagnosed with acoustic neuroma by means of an MRI (Figure 1). she also stressed that at about 18 months ago, after an emotional stress, she was affected by a sudden hearing loss episode in her left ear, when a skull CT scan was ordered for her, which showed a small hypodense area next to the right lateral ventricle horn (Figure 1), suggesting cerebral ischemic injury as the cause of the left-side sudden hearing loss. Audiology tests were carried out and upon tonal audiometry (Figure 1) we found a bilateral sensorineural hearing loss, and the patient still had a better hearing in her right ear. She was advised to wear a hearing aid in both ears and go through multidisciplinary outpatient follow up by a geriatrician, cardiologist and a neurologist.

## DISCUSSION

The case hereby reported is rare, it is a bilateral sudden hearing loss, of non-simultaneous occurrence, suggested by the tonal audiometry test^6^, as it shows bilateral sensorineural hearing loss above 30 dB in more than three contiguous frequencies^1^,^2^. Initially, she had right ear hearing loss together with tinnitus - a common symptom in 80% of the cases^4^,^5^, and the hearing loss cause was established by the MRI, which showed an acoustic neuroma. Later, she had sudden hearing loss on the left side, of probable vascular involvement, which showed in the skull CT scan^3^,^4^.

## FINAL REMARKS

The present report points to the likelihood of non-simultaneous sudden bilateral hearing loss, due to the participation of numerous etiological factors which, added together, may cause severe loss to the bearer. Thus, we stress the importance of the early diagnosis of hearing involvement and the control of pathologies, especially vascular ones, which may cause sudden hearing loss, as in this clinical case and thus have a negative repercussion in the quality of life of the elderly.
